# Supplementary figures and images for: Comparative whole-genome analyses of articular chondrocytes and skin fibroblasts reveal distinct genome instability landscapes in mesenchymal cell types
Source: bioRxiv. 2026 Feb 15:2026.02.13.705814. Preprint. [Version 1] doi: 10.64898/2026.02.13.705814 (PMC12918860; doi:10.64898/2026.02.13.705814)

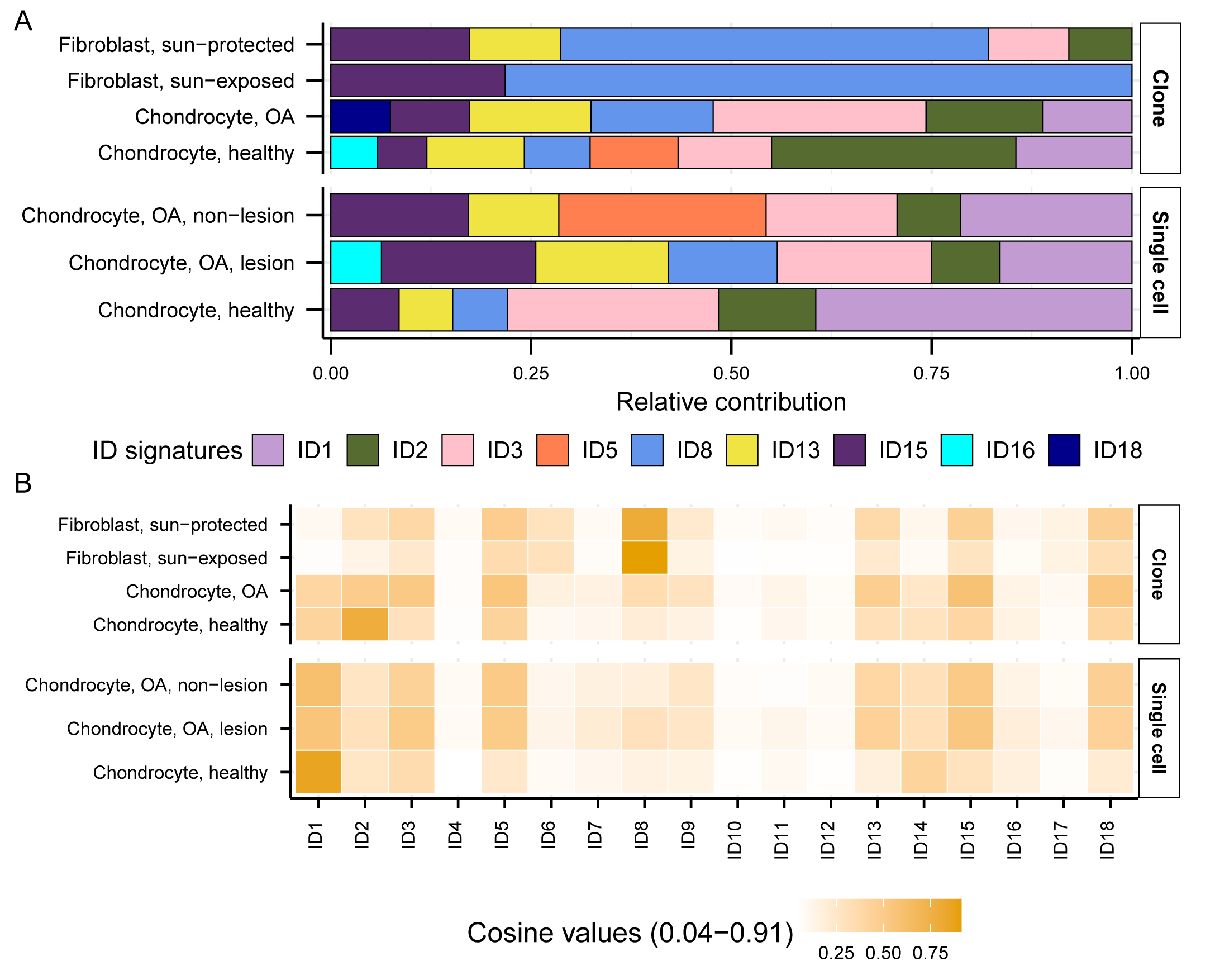

Supplement: Supplement 1 — Figure S1. Schema of chondrocyte colony isolation and DNA sequencing performed in this study. A. Bulk chondrocytes were isolated from donor cartilage tissues and seeded at low density to generate single-cell derived colonies. Colonies were propagated for less than 20 generations to isolate and sequence DNA. B. Consensus somatic variants (SNVs and InDels) from two callers were generated using bulk chondrocytes as the matched normal. Somatic calls were further filtered to retain high confidence clonal variants. Figure S2. Distribution of allele fraction of SNVs detected in chondrocyte samples sequenced in this study. Allele fractions of all SNVs before filtering are plotted in bins of five. Samples originating from the same donor are indicated by color. Figure S3. SNV load and accumulation rate per cell division in individual genomes. A. Total SNV load within each cell type is shown in boxplot. Each dot represents an individual sample. Asterisks represent statistical significance (Wilcoxon Rank Sum test) between connected cell types. Black solid connector lines indicate two-sided test, red dashed connector lines indicate one-sided test. *P value ≤ 0.05, **P value ≤ 0.01, ***P value ≤ 0.001. B. Donor mean mutation load plotted against donor age for each sub-group of cell type indicated. Correlation coefficient and one-sided p value from Spearman’s correlation analyses are indicated on each plot. Red line indicates best-fit linear regression. C. Mutation accumulation rate per cell division in different cell types shown in boxplot. Each dot represents an individual sample. Asterisks represent statistical significance (Wilcoxon Rank Sum test) between connected cell types. Black solid connector lines indicate two-sided test; red dashed connector lines indicate one-sided test. *P value ≤ 0.05, **P value ≤ 0.01, ***P value ≤ 0.001. All source data and p values from statistical analyses are available in supplemental table S2. Figure S4. Mutational profiles of COSMIC reference [file media-1.zip › Fig S11.tif]

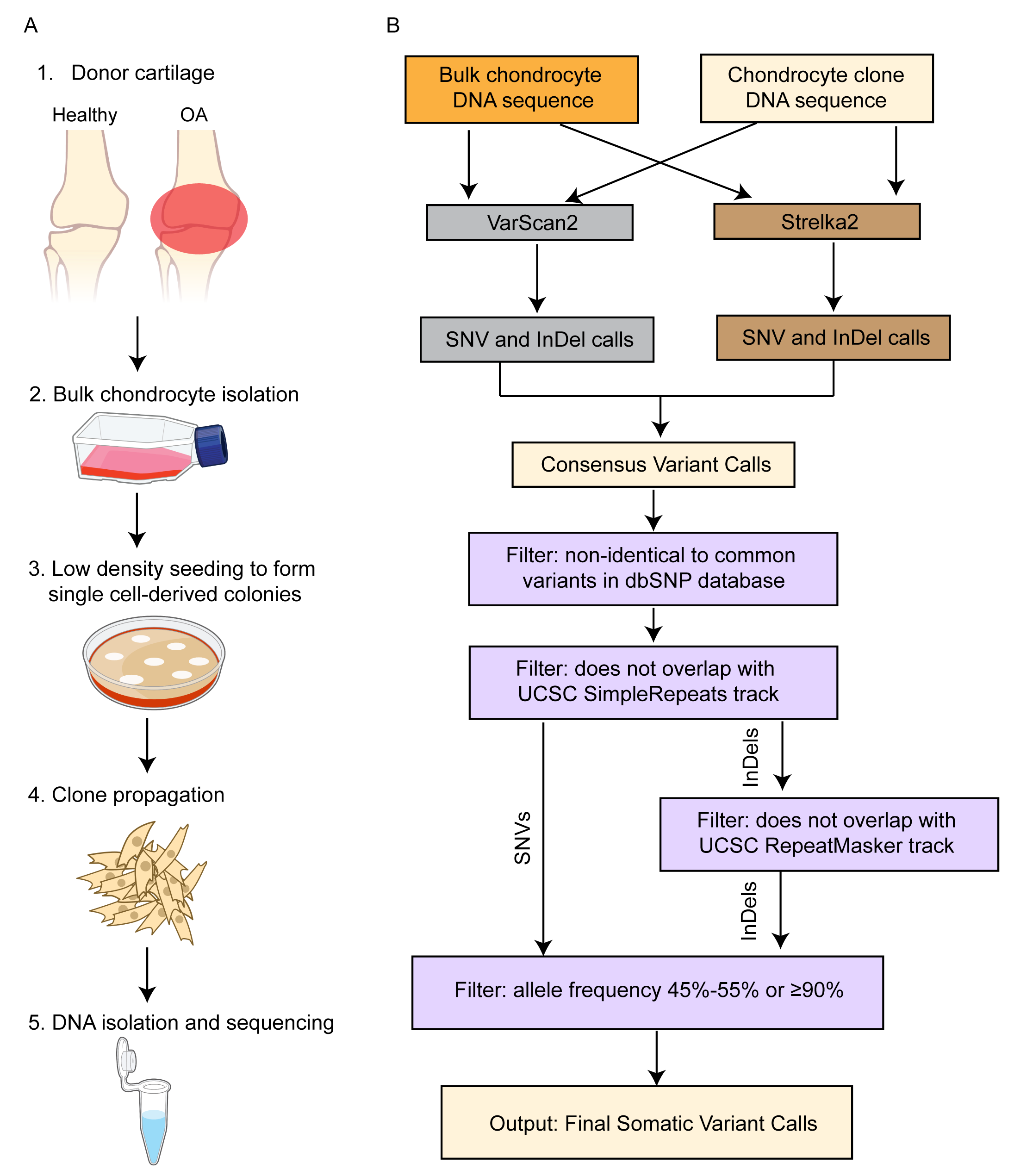

Supplement: Supplement 1 — Figure S1. Schema of chondrocyte colony isolation and DNA sequencing performed in this study. A. Bulk chondrocytes were isolated from donor cartilage tissues and seeded at low density to generate single-cell derived colonies. Colonies were propagated for less than 20 generations to isolate and sequence DNA. B. Consensus somatic variants (SNVs and InDels) from two callers were generated using bulk chondrocytes as the matched normal. Somatic calls were further filtered to retain high confidence clonal variants. Figure S2. Distribution of allele fraction of SNVs detected in chondrocyte samples sequenced in this study. Allele fractions of all SNVs before filtering are plotted in bins of five. Samples originating from the same donor are indicated by color. Figure S3. SNV load and accumulation rate per cell division in individual genomes. A. Total SNV load within each cell type is shown in boxplot. Each dot represents an individual sample. Asterisks represent statistical significance (Wilcoxon Rank Sum test) between connected cell types. Black solid connector lines indicate two-sided test, red dashed connector lines indicate one-sided test. *P value ≤ 0.05, **P value ≤ 0.01, ***P value ≤ 0.001. B. Donor mean mutation load plotted against donor age for each sub-group of cell type indicated. Correlation coefficient and one-sided p value from Spearman’s correlation analyses are indicated on each plot. Red line indicates best-fit linear regression. C. Mutation accumulation rate per cell division in different cell types shown in boxplot. Each dot represents an individual sample. Asterisks represent statistical significance (Wilcoxon Rank Sum test) between connected cell types. Black solid connector lines indicate two-sided test; red dashed connector lines indicate one-sided test. *P value ≤ 0.05, **P value ≤ 0.01, ***P value ≤ 0.001. All source data and p values from statistical analyses are available in supplemental table S2. Figure S4. Mutational profiles of COSMIC reference [file media-1.zip › Fig S1.tif]

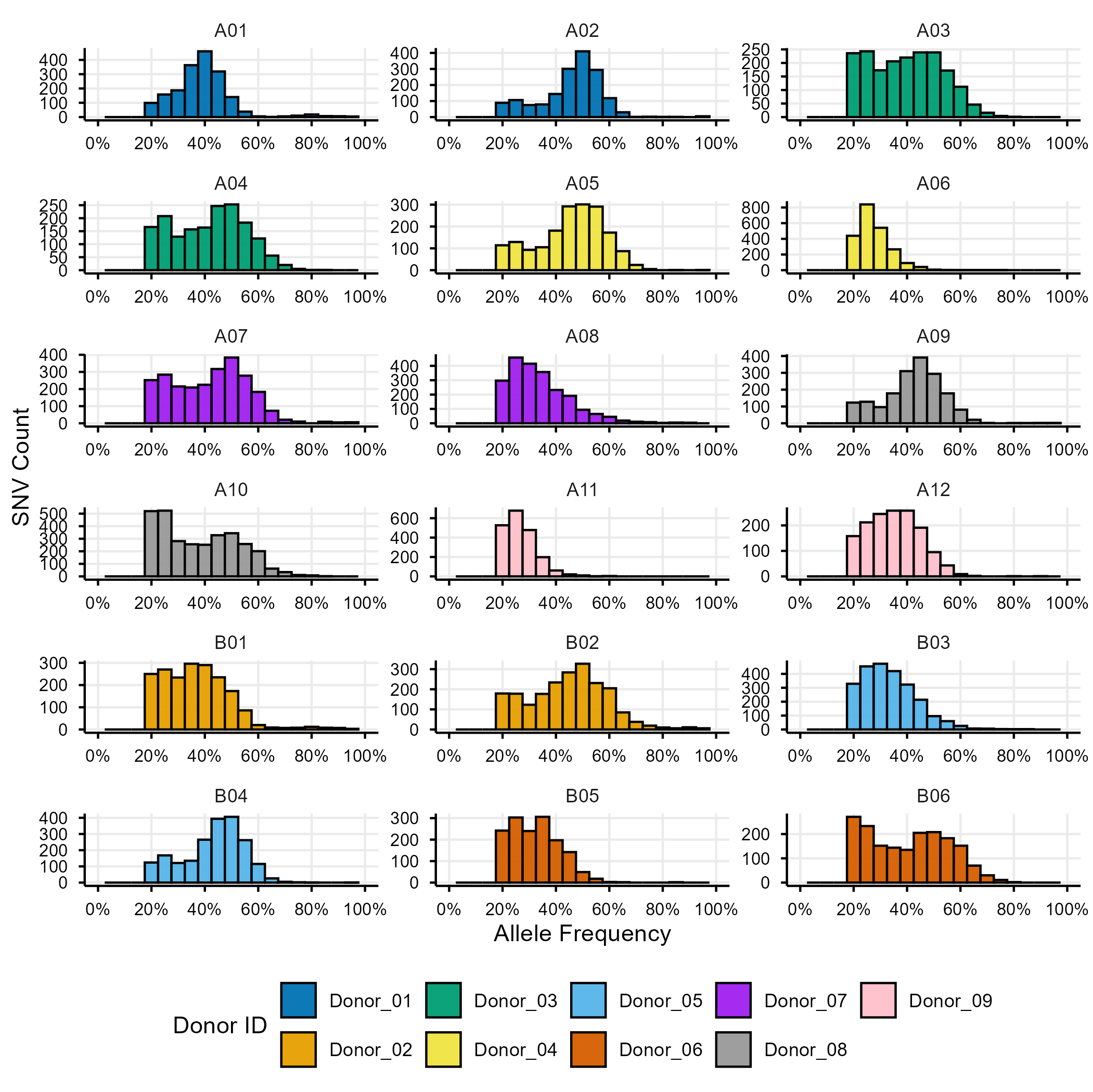

Supplement: Supplement 1 — Figure S1. Schema of chondrocyte colony isolation and DNA sequencing performed in this study. A. Bulk chondrocytes were isolated from donor cartilage tissues and seeded at low density to generate single-cell derived colonies. Colonies were propagated for less than 20 generations to isolate and sequence DNA. B. Consensus somatic variants (SNVs and InDels) from two callers were generated using bulk chondrocytes as the matched normal. Somatic calls were further filtered to retain high confidence clonal variants. Figure S2. Distribution of allele fraction of SNVs detected in chondrocyte samples sequenced in this study. Allele fractions of all SNVs before filtering are plotted in bins of five. Samples originating from the same donor are indicated by color. Figure S3. SNV load and accumulation rate per cell division in individual genomes. A. Total SNV load within each cell type is shown in boxplot. Each dot represents an individual sample. Asterisks represent statistical significance (Wilcoxon Rank Sum test) between connected cell types. Black solid connector lines indicate two-sided test, red dashed connector lines indicate one-sided test. *P value ≤ 0.05, **P value ≤ 0.01, ***P value ≤ 0.001. B. Donor mean mutation load plotted against donor age for each sub-group of cell type indicated. Correlation coefficient and one-sided p value from Spearman’s correlation analyses are indicated on each plot. Red line indicates best-fit linear regression. C. Mutation accumulation rate per cell division in different cell types shown in boxplot. Each dot represents an individual sample. Asterisks represent statistical significance (Wilcoxon Rank Sum test) between connected cell types. Black solid connector lines indicate two-sided test; red dashed connector lines indicate one-sided test. *P value ≤ 0.05, **P value ≤ 0.01, ***P value ≤ 0.001. All source data and p values from statistical analyses are available in supplemental table S2. Figure S4. Mutational profiles of COSMIC reference [file media-1.zip › Fig S2.tif]

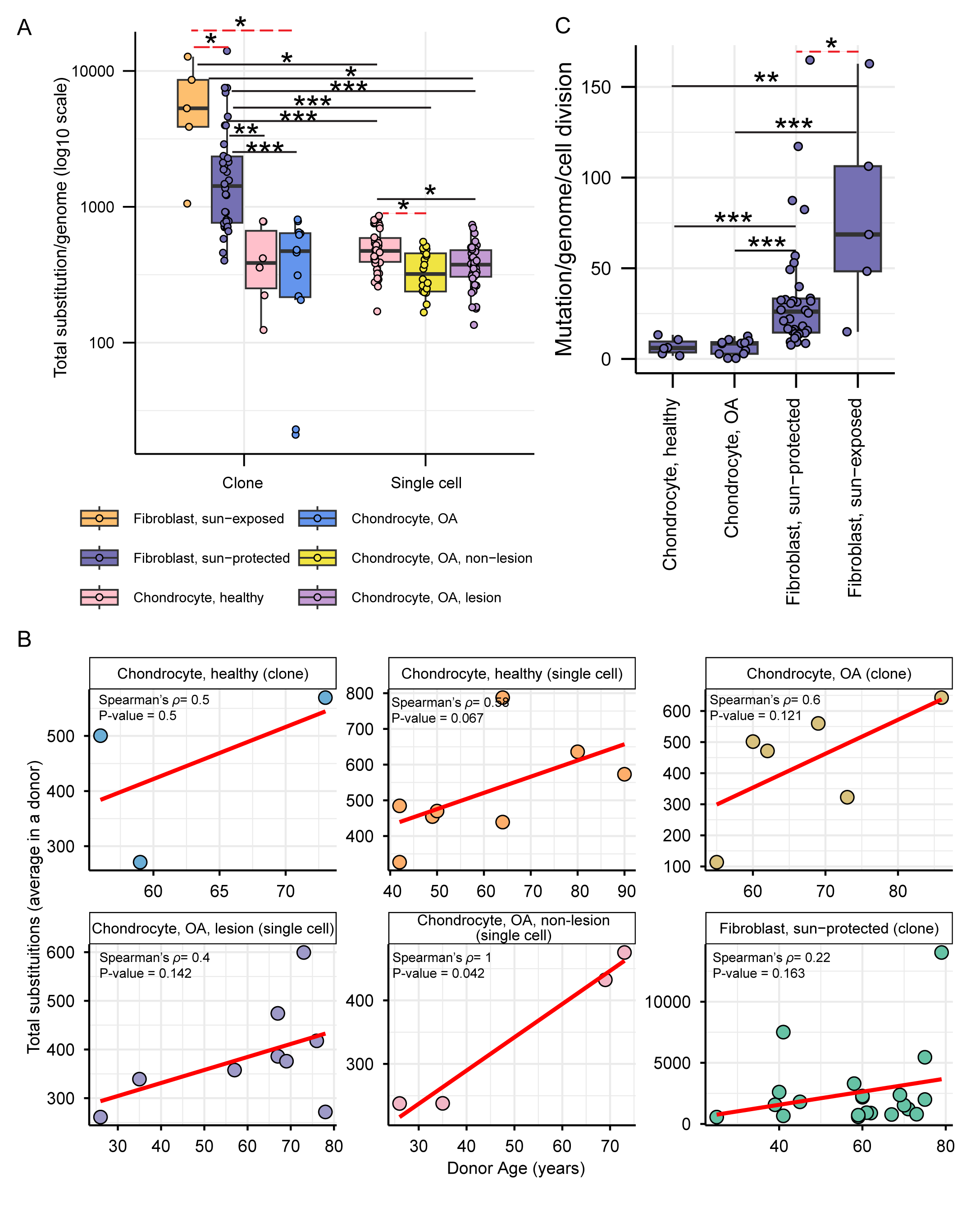

Supplement: Supplement 1 — Figure S1. Schema of chondrocyte colony isolation and DNA sequencing performed in this study. A. Bulk chondrocytes were isolated from donor cartilage tissues and seeded at low density to generate single-cell derived colonies. Colonies were propagated for less than 20 generations to isolate and sequence DNA. B. Consensus somatic variants (SNVs and InDels) from two callers were generated using bulk chondrocytes as the matched normal. Somatic calls were further filtered to retain high confidence clonal variants. Figure S2. Distribution of allele fraction of SNVs detected in chondrocyte samples sequenced in this study. Allele fractions of all SNVs before filtering are plotted in bins of five. Samples originating from the same donor are indicated by color. Figure S3. SNV load and accumulation rate per cell division in individual genomes. A. Total SNV load within each cell type is shown in boxplot. Each dot represents an individual sample. Asterisks represent statistical significance (Wilcoxon Rank Sum test) between connected cell types. Black solid connector lines indicate two-sided test, red dashed connector lines indicate one-sided test. *P value ≤ 0.05, **P value ≤ 0.01, ***P value ≤ 0.001. B. Donor mean mutation load plotted against donor age for each sub-group of cell type indicated. Correlation coefficient and one-sided p value from Spearman’s correlation analyses are indicated on each plot. Red line indicates best-fit linear regression. C. Mutation accumulation rate per cell division in different cell types shown in boxplot. Each dot represents an individual sample. Asterisks represent statistical significance (Wilcoxon Rank Sum test) between connected cell types. Black solid connector lines indicate two-sided test; red dashed connector lines indicate one-sided test. *P value ≤ 0.05, **P value ≤ 0.01, ***P value ≤ 0.001. All source data and p values from statistical analyses are available in supplemental table S2. Figure S4. Mutational profiles of COSMIC reference [file media-1.zip › Fig S3.tif]

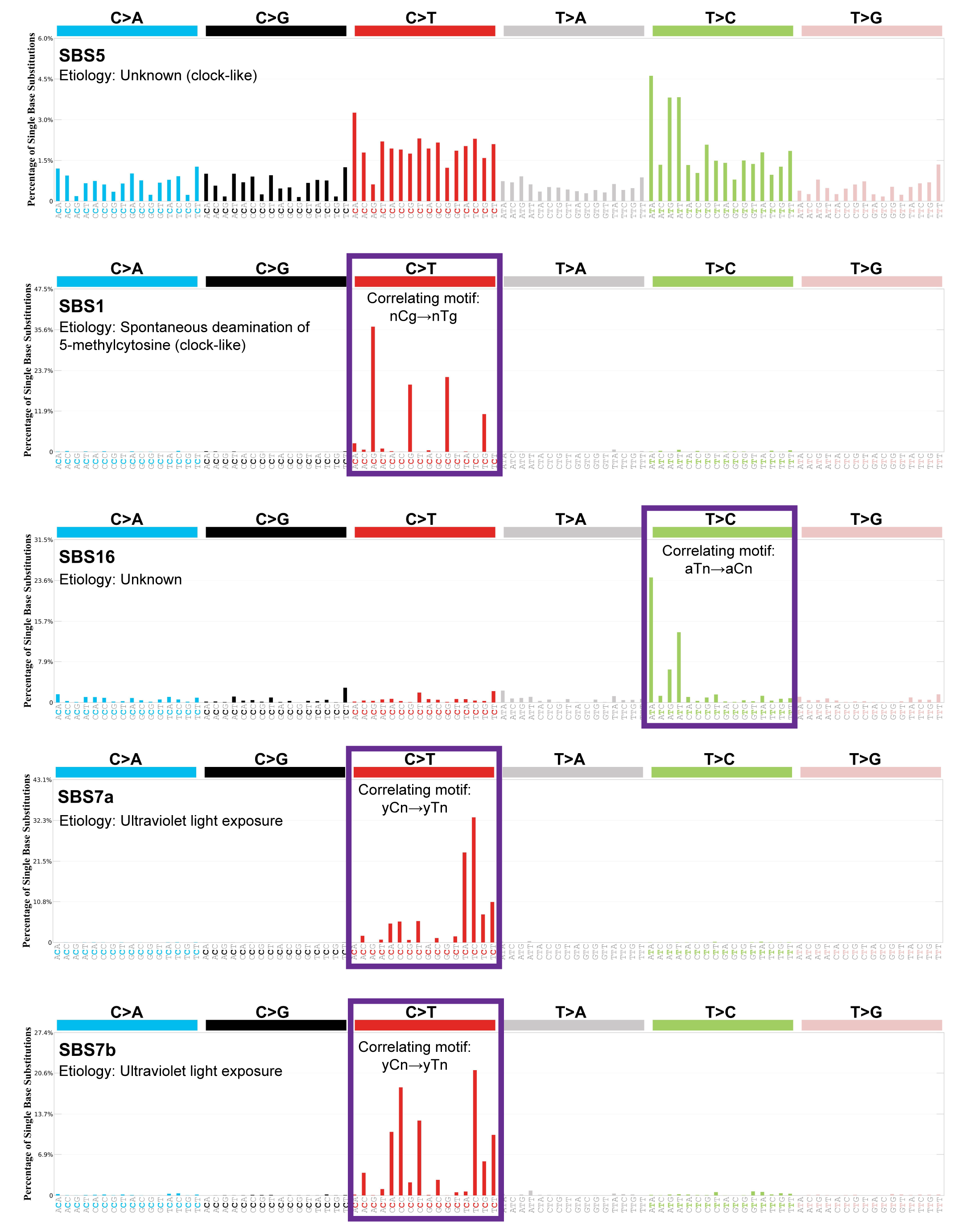

Supplement: Supplement 1 — Figure S1. Schema of chondrocyte colony isolation and DNA sequencing performed in this study. A. Bulk chondrocytes were isolated from donor cartilage tissues and seeded at low density to generate single-cell derived colonies. Colonies were propagated for less than 20 generations to isolate and sequence DNA. B. Consensus somatic variants (SNVs and InDels) from two callers were generated using bulk chondrocytes as the matched normal. Somatic calls were further filtered to retain high confidence clonal variants. Figure S2. Distribution of allele fraction of SNVs detected in chondrocyte samples sequenced in this study. Allele fractions of all SNVs before filtering are plotted in bins of five. Samples originating from the same donor are indicated by color. Figure S3. SNV load and accumulation rate per cell division in individual genomes. A. Total SNV load within each cell type is shown in boxplot. Each dot represents an individual sample. Asterisks represent statistical significance (Wilcoxon Rank Sum test) between connected cell types. Black solid connector lines indicate two-sided test, red dashed connector lines indicate one-sided test. *P value ≤ 0.05, **P value ≤ 0.01, ***P value ≤ 0.001. B. Donor mean mutation load plotted against donor age for each sub-group of cell type indicated. Correlation coefficient and one-sided p value from Spearman’s correlation analyses are indicated on each plot. Red line indicates best-fit linear regression. C. Mutation accumulation rate per cell division in different cell types shown in boxplot. Each dot represents an individual sample. Asterisks represent statistical significance (Wilcoxon Rank Sum test) between connected cell types. Black solid connector lines indicate two-sided test; red dashed connector lines indicate one-sided test. *P value ≤ 0.05, **P value ≤ 0.01, ***P value ≤ 0.001. All source data and p values from statistical analyses are available in supplemental table S2. Figure S4. Mutational profiles of COSMIC reference [file media-1.zip › Fig S4.tif]

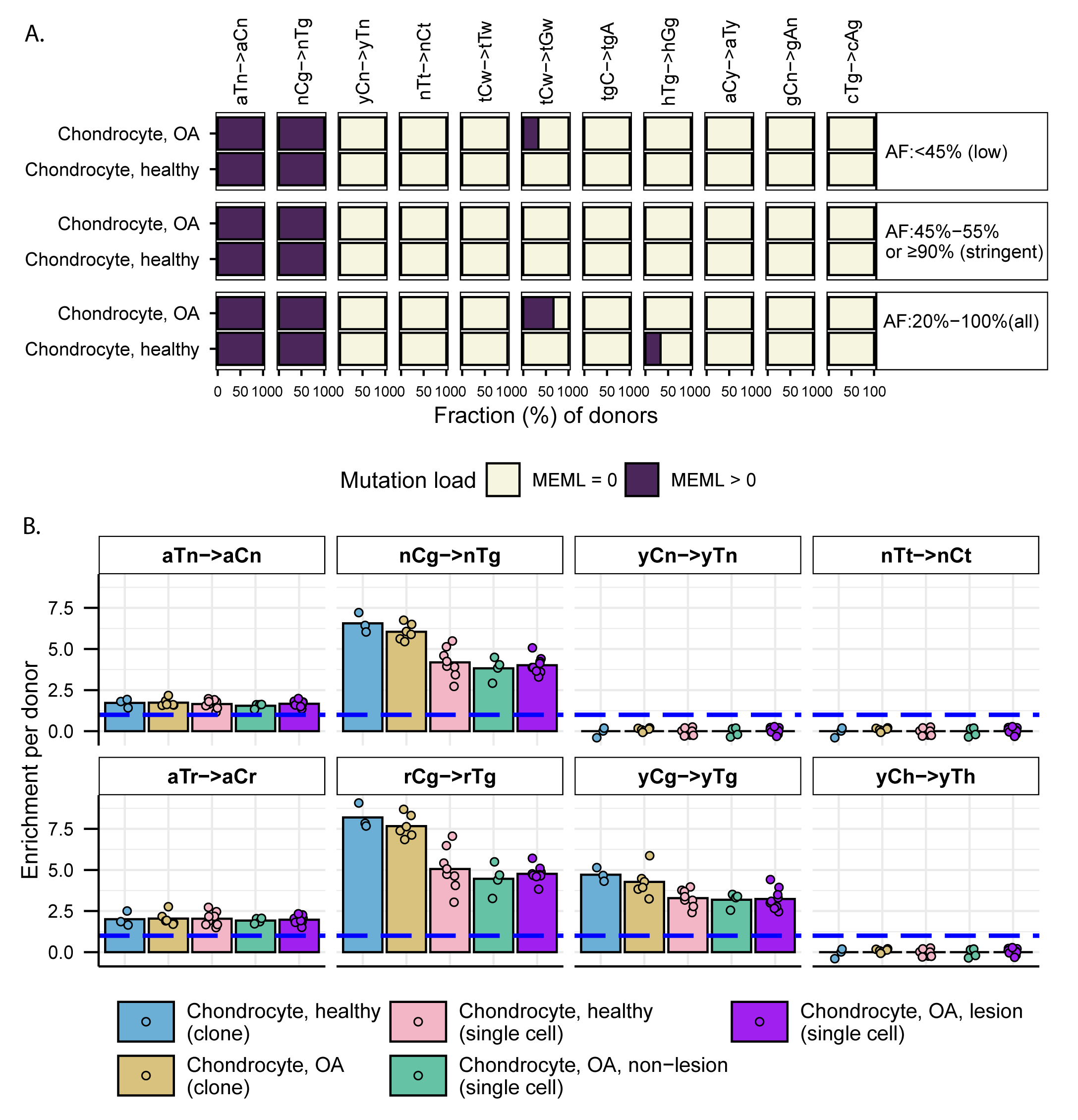

Supplement: Supplement 1 — Figure S1. Schema of chondrocyte colony isolation and DNA sequencing performed in this study. A. Bulk chondrocytes were isolated from donor cartilage tissues and seeded at low density to generate single-cell derived colonies. Colonies were propagated for less than 20 generations to isolate and sequence DNA. B. Consensus somatic variants (SNVs and InDels) from two callers were generated using bulk chondrocytes as the matched normal. Somatic calls were further filtered to retain high confidence clonal variants. Figure S2. Distribution of allele fraction of SNVs detected in chondrocyte samples sequenced in this study. Allele fractions of all SNVs before filtering are plotted in bins of five. Samples originating from the same donor are indicated by color. Figure S3. SNV load and accumulation rate per cell division in individual genomes. A. Total SNV load within each cell type is shown in boxplot. Each dot represents an individual sample. Asterisks represent statistical significance (Wilcoxon Rank Sum test) between connected cell types. Black solid connector lines indicate two-sided test, red dashed connector lines indicate one-sided test. *P value ≤ 0.05, **P value ≤ 0.01, ***P value ≤ 0.001. B. Donor mean mutation load plotted against donor age for each sub-group of cell type indicated. Correlation coefficient and one-sided p value from Spearman’s correlation analyses are indicated on each plot. Red line indicates best-fit linear regression. C. Mutation accumulation rate per cell division in different cell types shown in boxplot. Each dot represents an individual sample. Asterisks represent statistical significance (Wilcoxon Rank Sum test) between connected cell types. Black solid connector lines indicate two-sided test; red dashed connector lines indicate one-sided test. *P value ≤ 0.05, **P value ≤ 0.01, ***P value ≤ 0.001. All source data and p values from statistical analyses are available in supplemental table S2. Figure S4. Mutational profiles of COSMIC reference [file media-1.zip › Fig S5.tif]

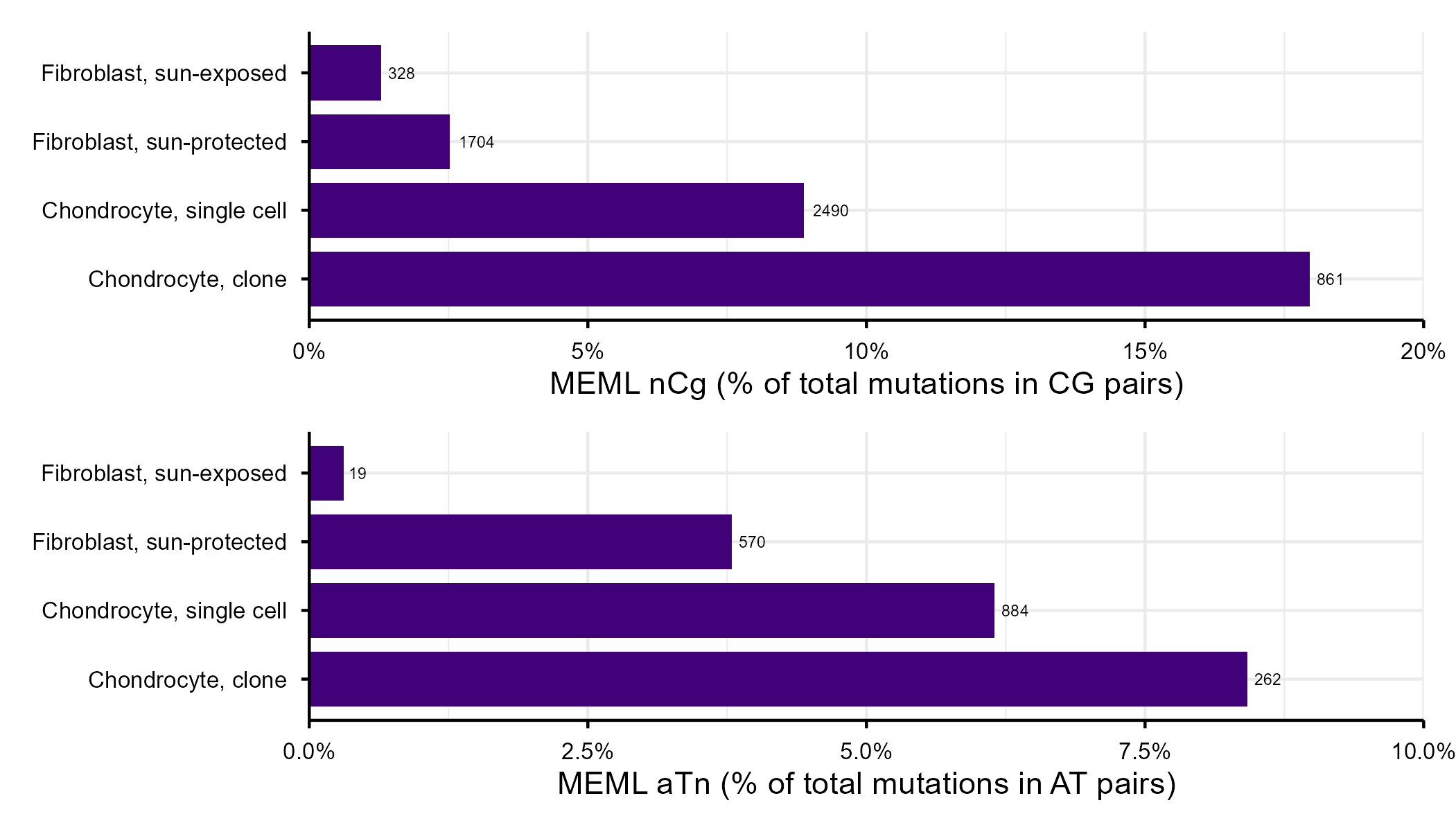

Supplement: Supplement 1 — Figure S1. Schema of chondrocyte colony isolation and DNA sequencing performed in this study. A. Bulk chondrocytes were isolated from donor cartilage tissues and seeded at low density to generate single-cell derived colonies. Colonies were propagated for less than 20 generations to isolate and sequence DNA. B. Consensus somatic variants (SNVs and InDels) from two callers were generated using bulk chondrocytes as the matched normal. Somatic calls were further filtered to retain high confidence clonal variants. Figure S2. Distribution of allele fraction of SNVs detected in chondrocyte samples sequenced in this study. Allele fractions of all SNVs before filtering are plotted in bins of five. Samples originating from the same donor are indicated by color. Figure S3. SNV load and accumulation rate per cell division in individual genomes. A. Total SNV load within each cell type is shown in boxplot. Each dot represents an individual sample. Asterisks represent statistical significance (Wilcoxon Rank Sum test) between connected cell types. Black solid connector lines indicate two-sided test, red dashed connector lines indicate one-sided test. *P value ≤ 0.05, **P value ≤ 0.01, ***P value ≤ 0.001. B. Donor mean mutation load plotted against donor age for each sub-group of cell type indicated. Correlation coefficient and one-sided p value from Spearman’s correlation analyses are indicated on each plot. Red line indicates best-fit linear regression. C. Mutation accumulation rate per cell division in different cell types shown in boxplot. Each dot represents an individual sample. Asterisks represent statistical significance (Wilcoxon Rank Sum test) between connected cell types. Black solid connector lines indicate two-sided test; red dashed connector lines indicate one-sided test. *P value ≤ 0.05, **P value ≤ 0.01, ***P value ≤ 0.001. All source data and p values from statistical analyses are available in supplemental table S2. Figure S4. Mutational profiles of COSMIC reference [file media-1.zip › Fig S6.tif]

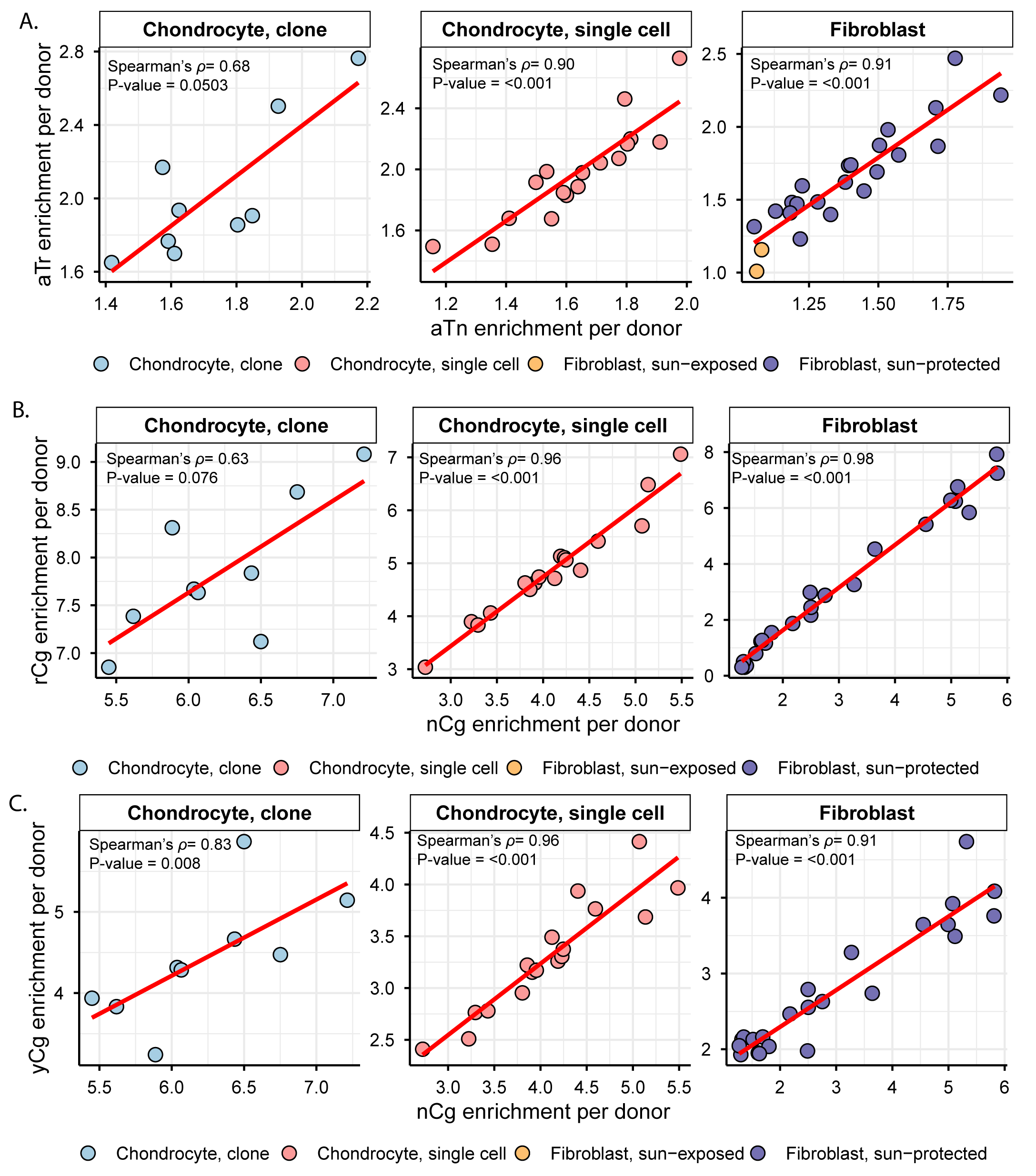

Supplement: Supplement 1 — Figure S1. Schema of chondrocyte colony isolation and DNA sequencing performed in this study. A. Bulk chondrocytes were isolated from donor cartilage tissues and seeded at low density to generate single-cell derived colonies. Colonies were propagated for less than 20 generations to isolate and sequence DNA. B. Consensus somatic variants (SNVs and InDels) from two callers were generated using bulk chondrocytes as the matched normal. Somatic calls were further filtered to retain high confidence clonal variants. Figure S2. Distribution of allele fraction of SNVs detected in chondrocyte samples sequenced in this study. Allele fractions of all SNVs before filtering are plotted in bins of five. Samples originating from the same donor are indicated by color. Figure S3. SNV load and accumulation rate per cell division in individual genomes. A. Total SNV load within each cell type is shown in boxplot. Each dot represents an individual sample. Asterisks represent statistical significance (Wilcoxon Rank Sum test) between connected cell types. Black solid connector lines indicate two-sided test, red dashed connector lines indicate one-sided test. *P value ≤ 0.05, **P value ≤ 0.01, ***P value ≤ 0.001. B. Donor mean mutation load plotted against donor age for each sub-group of cell type indicated. Correlation coefficient and one-sided p value from Spearman’s correlation analyses are indicated on each plot. Red line indicates best-fit linear regression. C. Mutation accumulation rate per cell division in different cell types shown in boxplot. Each dot represents an individual sample. Asterisks represent statistical significance (Wilcoxon Rank Sum test) between connected cell types. Black solid connector lines indicate two-sided test; red dashed connector lines indicate one-sided test. *P value ≤ 0.05, **P value ≤ 0.01, ***P value ≤ 0.001. All source data and p values from statistical analyses are available in supplemental table S2. Figure S4. Mutational profiles of COSMIC reference [file media-1.zip › Fig S7.tif]

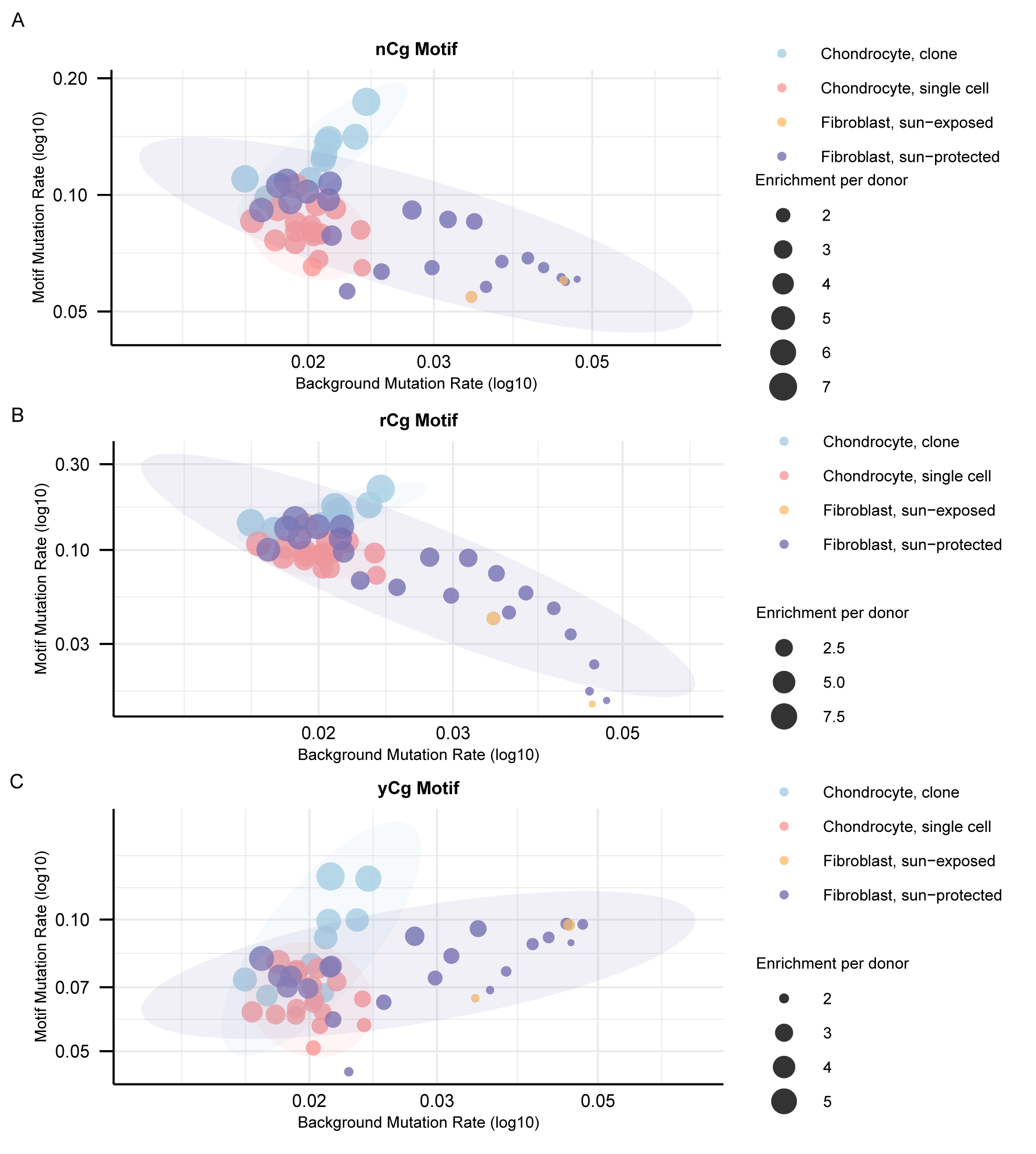

Supplement: Supplement 1 — Figure S1. Schema of chondrocyte colony isolation and DNA sequencing performed in this study. A. Bulk chondrocytes were isolated from donor cartilage tissues and seeded at low density to generate single-cell derived colonies. Colonies were propagated for less than 20 generations to isolate and sequence DNA. B. Consensus somatic variants (SNVs and InDels) from two callers were generated using bulk chondrocytes as the matched normal. Somatic calls were further filtered to retain high confidence clonal variants. Figure S2. Distribution of allele fraction of SNVs detected in chondrocyte samples sequenced in this study. Allele fractions of all SNVs before filtering are plotted in bins of five. Samples originating from the same donor are indicated by color. Figure S3. SNV load and accumulation rate per cell division in individual genomes. A. Total SNV load within each cell type is shown in boxplot. Each dot represents an individual sample. Asterisks represent statistical significance (Wilcoxon Rank Sum test) between connected cell types. Black solid connector lines indicate two-sided test, red dashed connector lines indicate one-sided test. *P value ≤ 0.05, **P value ≤ 0.01, ***P value ≤ 0.001. B. Donor mean mutation load plotted against donor age for each sub-group of cell type indicated. Correlation coefficient and one-sided p value from Spearman’s correlation analyses are indicated on each plot. Red line indicates best-fit linear regression. C. Mutation accumulation rate per cell division in different cell types shown in boxplot. Each dot represents an individual sample. Asterisks represent statistical significance (Wilcoxon Rank Sum test) between connected cell types. Black solid connector lines indicate two-sided test; red dashed connector lines indicate one-sided test. *P value ≤ 0.05, **P value ≤ 0.01, ***P value ≤ 0.001. All source data and p values from statistical analyses are available in supplemental table S2. Figure S4. Mutational profiles of COSMIC reference [file media-1.zip › Fig S8.tif]

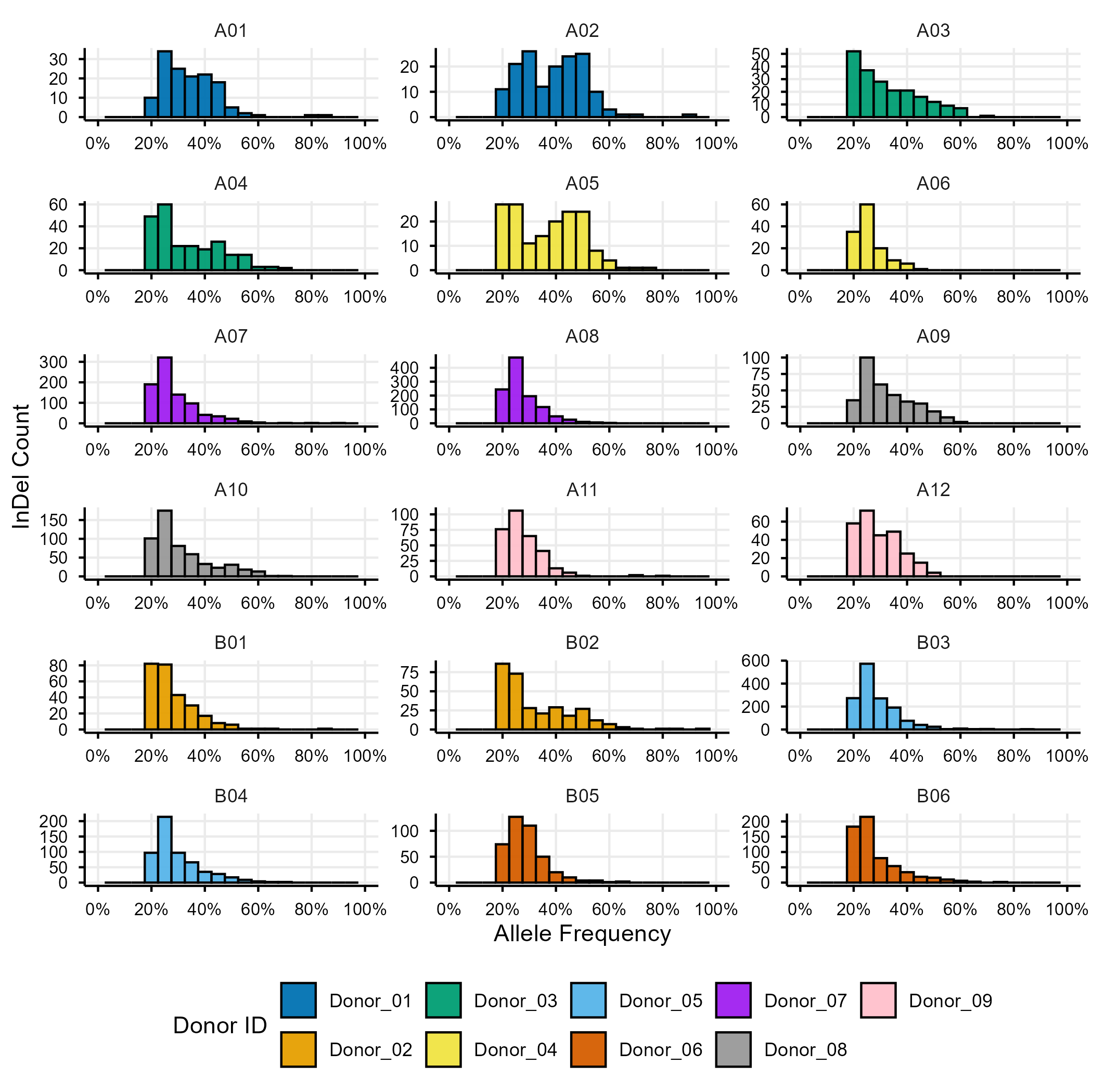

Supplement: Supplement 1 — Figure S1. Schema of chondrocyte colony isolation and DNA sequencing performed in this study. A. Bulk chondrocytes were isolated from donor cartilage tissues and seeded at low density to generate single-cell derived colonies. Colonies were propagated for less than 20 generations to isolate and sequence DNA. B. Consensus somatic variants (SNVs and InDels) from two callers were generated using bulk chondrocytes as the matched normal. Somatic calls were further filtered to retain high confidence clonal variants. Figure S2. Distribution of allele fraction of SNVs detected in chondrocyte samples sequenced in this study. Allele fractions of all SNVs before filtering are plotted in bins of five. Samples originating from the same donor are indicated by color. Figure S3. SNV load and accumulation rate per cell division in individual genomes. A. Total SNV load within each cell type is shown in boxplot. Each dot represents an individual sample. Asterisks represent statistical significance (Wilcoxon Rank Sum test) between connected cell types. Black solid connector lines indicate two-sided test, red dashed connector lines indicate one-sided test. *P value ≤ 0.05, **P value ≤ 0.01, ***P value ≤ 0.001. B. Donor mean mutation load plotted against donor age for each sub-group of cell type indicated. Correlation coefficient and one-sided p value from Spearman’s correlation analyses are indicated on each plot. Red line indicates best-fit linear regression. C. Mutation accumulation rate per cell division in different cell types shown in boxplot. Each dot represents an individual sample. Asterisks represent statistical significance (Wilcoxon Rank Sum test) between connected cell types. Black solid connector lines indicate two-sided test; red dashed connector lines indicate one-sided test. *P value ≤ 0.05, **P value ≤ 0.01, ***P value ≤ 0.001. All source data and p values from statistical analyses are available in supplemental table S2. Figure S4. Mutational profiles of COSMIC reference [file media-1.zip › Fig S9.tif]

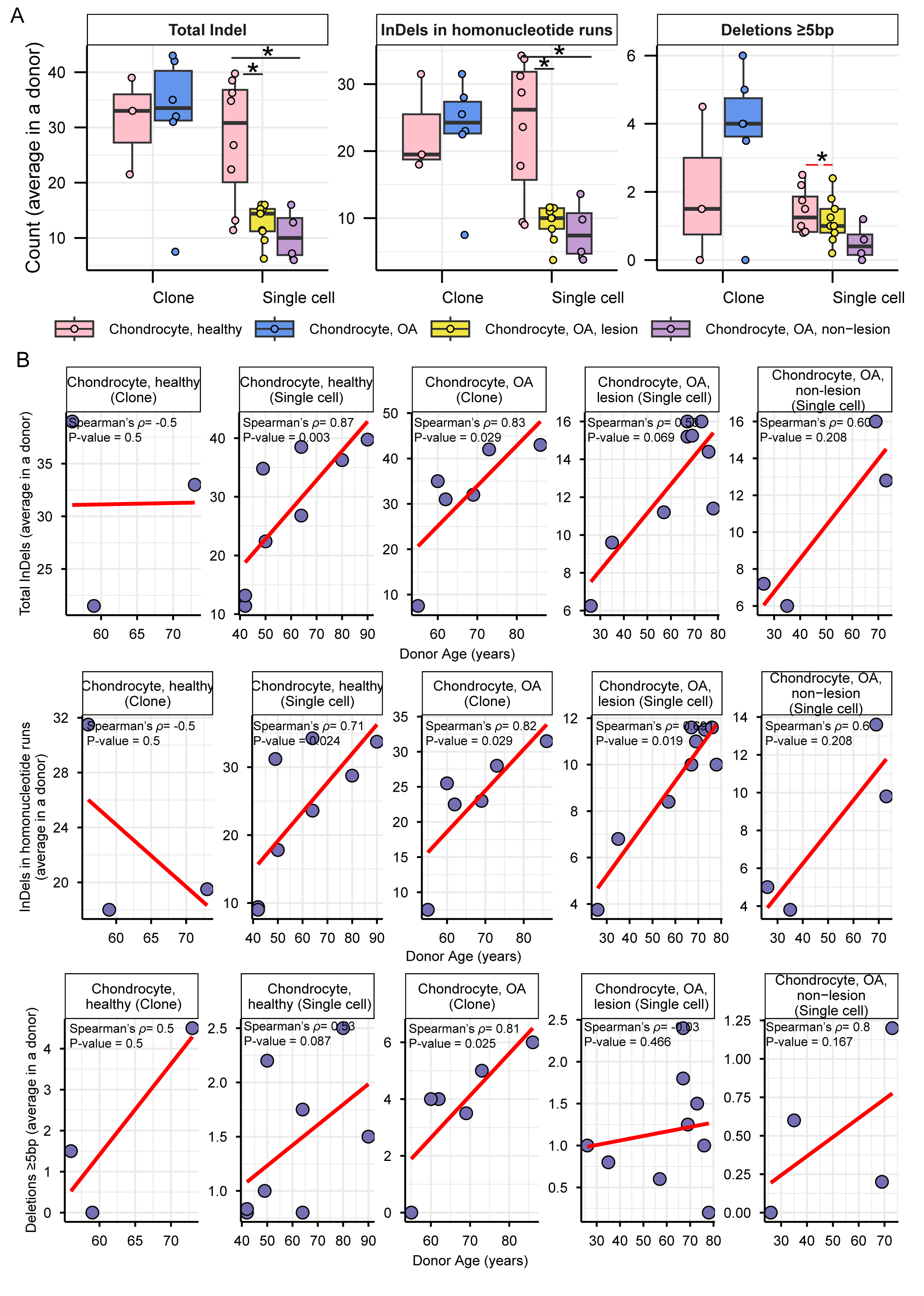

Supplement: Supplement 1 — Figure S1. Schema of chondrocyte colony isolation and DNA sequencing performed in this study. A. Bulk chondrocytes were isolated from donor cartilage tissues and seeded at low density to generate single-cell derived colonies. Colonies were propagated for less than 20 generations to isolate and sequence DNA. B. Consensus somatic variants (SNVs and InDels) from two callers were generated using bulk chondrocytes as the matched normal. Somatic calls were further filtered to retain high confidence clonal variants. Figure S2. Distribution of allele fraction of SNVs detected in chondrocyte samples sequenced in this study. Allele fractions of all SNVs before filtering are plotted in bins of five. Samples originating from the same donor are indicated by color. Figure S3. SNV load and accumulation rate per cell division in individual genomes. A. Total SNV load within each cell type is shown in boxplot. Each dot represents an individual sample. Asterisks represent statistical significance (Wilcoxon Rank Sum test) between connected cell types. Black solid connector lines indicate two-sided test, red dashed connector lines indicate one-sided test. *P value ≤ 0.05, **P value ≤ 0.01, ***P value ≤ 0.001. B. Donor mean mutation load plotted against donor age for each sub-group of cell type indicated. Correlation coefficient and one-sided p value from Spearman’s correlation analyses are indicated on each plot. Red line indicates best-fit linear regression. C. Mutation accumulation rate per cell division in different cell types shown in boxplot. Each dot represents an individual sample. Asterisks represent statistical significance (Wilcoxon Rank Sum test) between connected cell types. Black solid connector lines indicate two-sided test; red dashed connector lines indicate one-sided test. *P value ≤ 0.05, **P value ≤ 0.01, ***P value ≤ 0.001. All source data and p values from statistical analyses are available in supplemental table S2. Figure S4. Mutational profiles of COSMIC reference [file media-1.zip › Fig S10.tif]
